# The influence of educational transitions on loneliness and mental health from emerging adults’ perspectives

**DOI:** 10.1080/17482631.2024.2422142

**Published:** 2024-10-27

**Authors:** Amanda Jasmin Emilia Sundqvist, Pia Nyman-Kurkiala, Ottar Ness, Jessica Hemberg

**Affiliations:** a Department of Health Sciences, Faculty of Education and Welfare Studies, Åbo Akademi University, Vaasa, Finland; b Department of Education and Lifelong Learning, Faculty of Social and Educational Sciences, Norwegian University of Science and Technology (NTNU), Dragvoll, Norway

**Keywords:** Education, loneliness, mental health, transitions, young people, qualitative

## Abstract

Educational transitions can influence loneliness and increase mental health issues for young people transitioning between educational stages. The aim was to explore the link between educational transitions and emerging adults’ loneliness and mental health and how they perceive they can alleviate and/or improve these issues. Semi-structured qualitative interviews with a total of 12 emerging adults, 10 females and two males aged 18–29 (mean age, 22.91) were conducted. Data were analysed using qualitative content analysis. Emerging adults’ loneliness and mental health across educational transitions could be influenced by changes to their sense of security, for example concurrent transitions, increased academic demands, changed social network, insufficient attachment, lack of community, or changed physical environment. Transitions can be associated with positive expectations and self-development but also worry, emotional turmoil, loneliness, and mental health issues. A better transition experience can be enabled by increasing resources and activities and promoting a secure environment.

## Introduction

A substantial proportion of individuals in many countries worldwide experience loneliness (Surkalim et al., 2022). Loneliness is considered a public health concern due to its detrimental effects on mental and physical health (Goosby et al., 2013; Hards et al., 2022; Luanaigh & Lawlor, 2008) as well as overall well-being (Goodfellow et al., 2022). Loneliness can be defined as experiencing deficiencies in one’s qualitative and/or quantitative social relationships (Perlman & Peplau, 1998). Mental health can be defined as a state of well-being where there is not just an absence of mental illness but also a dynamic state of internal balance, allowing a person to use their abilities in harmony with society’s universal values (Galderisi et al., 2015).

Researchers have found that loneliness is commonly experienced among young people (Jefferson et al., 2023; Qualter et al., 2015; Shovestul et al., 2020), who report higher levels of loneliness than the elderly (Barreto et al., 2021; Heinrich & Gullone, 2006). Loneliness is particularly common during emerging adulthood (ages 18–30) (Luhmann & Hawkley, 2016; Shovestul et al., 2020) and peaks among the youngest in higher education (18–20/21-year-olds) (Hysing et al., 2020; Zahedi et al., 2022), possibly due to the upper secondary/high school to university transition (Hysing et al., 2020). Additionally, the onset of most mental disorders occurs during childhood, adolescence, or the early adult years (de Girolamo et al., 2012; Kessler et al., 2007), with around 75% starting before the age of 24 (Fusar-Poli, 2019). Untreated loneliness can have serious mental and physical health consequences, including chronic stress, poor sleep quality, suicidal ideation (Mushtaq et al., 2014), depression (Hutten et al., 2021; Mushtaq et al., 2014), anxiety, somatic symptoms (Hutten et al., 2021), psychological health complaints (Lyyra et al., 2022), high cholesterol levels, diabetes, psychological distress, and/or poor self-perceived health (Richard et al., 2017).

### Educational transitions and their relation to loneliness and mental health

Emerging adulthood involves frequent change, instability, and the exploration of different life directions (Arnett, 2000). These shifts, combined with changes in the social environment, can increase rates of loneliness (Qualter et al., 2015). Young people often experience social changes during educational transitions, such as new peers, different learning structures, a lower social status (Blakemore & Mills, 2014), moving from a familiar to an unfamiliar and unpredictable context (Benner, 2011), shifting from a smaller school setting to a larger one, facing new teachers, and/or larger class sizes (Eccles & Roeser, 2011). Such changes can disrupt social networks and introduce new demands (Mikal et al., 2013), potentially causing concerns about social acceptance (Hanewald, 2013). While these changes might be positive for some, they might also be stressful and traumatic, negatively impacting well-being (Jindal-Snape, 2016).

Researchers in a study set in Poland have found that 42.3% of upper secondary/high school students reported moderate loneliness, 6.67% severe loneliness, and 25% experienced anxiety or depression (Dziedzic et al., 2021). Also, the transition to upper secondary/high school can lead to increased anxiety, loneliness, depression, and a decline in self-worth. Moving to a larger school may disrupt existing social networks, introduce new peers, lead to disruptions or changes with school personnel and educators, and involve interacting with older students, which can increase peer stressors and potentially heighten levels of depression (Benner, 2011). Additionally, students often report receiving less support from upper secondary/high school teachers and administrators compared to those in lower secondary/middle school (Barber & Olsen, 2004; Benner, 2011), while they also report concerns about competence, social belongingness, and control (Sundqvist et al., 2024).

Loneliness has been seen to be a significant issue among higher education students (Zahedi et al., 2022), with both emotional and social loneliness linked to anxiety and depression (Diehl et al., 2018). In Finland, about 28% of students reported mental health problems related to ongoing stress, unhappiness, concentration difficulties, and sleep loss, with 30% experiencing considerable stress (Kunttu & Pesonen, 2012). In Norway, 14–24% of students often felt isolated, lacked companionship, or felt left out (Hysing et al., 2020). In Germany, 14.2% reported depressive symptoms, 16.3% anxiety symptoms (Wörfel et al., 2016), 32.4% moderate loneliness, and 3.2% severe loneliness, with emotional loneliness being more prevalent than social loneliness (Diehl et al., 2018).

Researchers have found that starting higher education in a new location can be a vulnerable time, often increasing loneliness due to separation from existing social networks and the need to adapt to new environments and social circles (Hemberg et al., 2022; Rönkä et al., 2018; Sundqvist & Hemberg, 2021). The transition to higher education often requires adapting to new lifestyles and responsibilities. Difficulties in communication and a lack of social connections during this period can increase loneliness (Fardghassemi et al., 2022). While some students feel excited about new experiences, others struggle with the increased demands and feel unprepared, leading to social and emotional loneliness (Hemberg et al., 2022). Moving to a new location can also be challenging and is linked to loneliness at the start of higher education studies (Jaud et al., 2023). Psychological well-being often decreases during the first semester of higher education, with distress peaking early and not fully returning to pre-registration levels (Bewick et al., 2010).

### The Finnish education system

In Finland, young people attend comprehensive school (primary/lower secondary education) from ages 7 to 17, followed by three years of upper secondary/high school, either general or vocational, with the option to leave school at 18. After upper secondary/high school, students can apply to universities and attain Bachelor’s, Master’s, or Doctoral degrees. Universities of applied sciences provide practice-oriented Bachelor’s degrees and Master’s degrees after two years of work experience (Ministry of Education and Culture, n.d.).

### Transitions as a concept in nursing

In nursing, transitions can be defined as a passage from one life phase to another or a movement from one condition or status to another (Schumacher & Meleis, 1994), when a person seeks to adapt to a change, event, or disruption (Kralik et al., 2006). Transitions usually involve adjustments in roles, relationships, identities, or other aspects of life (Schumacher & Meleis, 1994). Transitions can be anticipated or unanticipated and may occur over varying timescales (Chick & Meleis, 1986). They often require psychological adaptation as individuals adjust to new circumstances (Kralik et al., 2006). Transitions usually entail life changes, which can impact health and well-being (Schumacher & Meleis, 1994). Transitions might also involve a sense of “disconnectedness” due to disruptions in previously stable aspects of life. The process of adjustment can vary in pace, occurring slowly or more rapidly (Chick & Meleis, 1986).

## Aim of the study

The aim was to explore the link between educational transitions and emerging adults’ loneliness and mental health and how they perceive they can alleviate and/or improve these issues. The research questions were: How do educational transitions link to emerging adults’ loneliness and mental health? What are emerging adults’ perceptions of how increases in loneliness and decline in mental health linked to educational transitions can be alleviated?

## Materials and methods

A qualitative approach was used. The Consolidated criteria for reporting qualitative research (COREQ) checklist were used as a guideline for reporting the study (Tong et al., 2007).

### Sampling, setting, and recruitment

A combination of purposeful, self-selection, and snowball sampling was used. Purposeful sampling involves selecting participants with knowledge and experience relevant to the study focus (Palinkas et al., 2015). Participants were chosen based on specific characteristics that aligned with the purpose of the study, with inclusion criteria targeting emerging adults aged 17–29 who had experienced loneliness or a decline in mental health during the transition to upper secondary/high school or higher education. No exclusion was made based on previous psychiatric diagnoses. Gender balance was sought and only Swedish-speaking participants were included because the study was set in a region where Swedish is the primary language. Participants with prior relationships with the researchers were excluded.

Self-selection sampling was realized by allowing interested individuals to sign up for the study based on own willingness to participate (Galloway, 2005). This method was selected to maintain participants’ autonomy and comfort, especially given the sensitive nature of the topic. The study was conducted at an upper secondary/high school and a university in Finland. Information about the study was distributed to students through email, flyers, and social media (Facebook, Instagram), with flyers physically placed out at the included institutions. A presentation about the study was given by the first researcher during courses at an upper secondary/high school. The inclusion criteria were outlined in the emails and flyers, and interested individuals were invited to contact the researchers for more details. Those who met the inclusion criteria were eligible to participate in the study. Snowball sampling was even used, where initial participants were asked to refer others who met the study criteria and might be interested in participating (Omona, 2013).

### Data collection and analysis

Semi-structured interviews were used. Semi-structured interviews can be used to examine a pre-defined subject where the researcher is aware of some areas of interest but also wishes to examine new perspectives (Newing, 2010). The first researcher (MSc, PhD student, female) created an interview guide, which was reviewed and critiqued by the second, third, and fourth researchers. The guide was refined as interviews progressed. The first researcher conducted all interviews, either in person at the research institution or through video conference. Participants were informed of the study aim beforehand. Interviews were audio-recorded, transcribed verbatim, and supplemented with field notes. Interview durations ranged from 37 to 155 minutes. A total of 785 minutes was spent interviewing all participants, with an average of 65.38 minutes per interview.

A total of 12 emerging adults, 10 females and two males aged 18–29 (mean age: 22.91), participated in the study. Two participants were enrolled in upper secondary/high school and ten were enrolled in higher education. Ten (83.33%) participants had moved location to start higher education. For an overview of the participant characteristics, see Table I.

**Table I.** Overview of the participant characteristics.

Participant	Age	Gender	Marital status	Accommodation	Current education level
P01	24	Woman	In relationship	Cohabitant	Higher education
P02	26	Man	In relationship	Own apartment	Higher education
P03	26	Woman	Single	Own apartment	Higher education
P04	24	Woman	In relationship	Own apartment	Higher education
P05	29	Woman	In relationship	Cohabitant	Higher education
P06	22	Man	In relationship	Own apartment	Higher education
P07	19	Woman	Single	Own apartment	Higher education
P08	20	Woman	Single	Cohabitant	Higher education
P09	28	Woman	Married	Cohabitant	Higher education
P10	18	Woman	Single	Cohabitant	Upper secondary school/high school
P11	18	Woman	In relationship	Cohabitant	Upper secondary school/high school
P12	21	Woman	Single	Own apartment	Higher education

The principle of data saturation was used during the data collection phase, i.e., data were collected until it was determined that the data were adequate and comprehensive and no additional information could be obtained (Morse, 1995). Strategic sampling ensured a cohesive sample with shared characteristics related to the transition to upper secondary/high school or university. Open-ended questions provided detailed and in-depth data. These included: What kind of feelings arose during the transition? Have you experienced loneliness or deterioration of your mental health during a transition? If yes, can you tell me more about it? How did it feel and what triggered the feelings? If you have experienced loneliness or deterioration of your mental health during a transition, what do you think was the cause of it? Which factors contributed to it? What could be done to facilitate transitions in your opinion? What kind of support would you have preferred? Did you feel that any (form of) support was lacking?

The analysis was conducted using the NVivo transcription program (Version 13) (Lumivero, 2020). Data were analysed using qualitative content analysis inspired by Graneheim and Lundman (2004). The first author primarily conducted the analysis. Familiarization of data was reached by reading the interviews several times. Whole interviews in which educational transitions in relation to loneliness and mental health were explored. Meaning units were extracted from those interviews and condensed; the text was shortened while still preserving its meaning and later abstracted and labelled as codes. The codes were then grouped into subcategories based on their similarities and differences and the subcategories then sorted into categories. Lastly, themes were formulated. The codes, categories, and themes were reviewed by all authors and discussed until consensus was reached. For an example of the data analysis, see Table II.

**Table II.** Example of the data analysis.

Meaning unit	Condensed meaning unit	Code	Subcategory	Category
… I somehow felt that I lost control … over … I had such a feeling of control earlier during the summer … and I had been on a trip with my car and everything had gone so well … and then all of a sudden I start studying and lose that sense of control …	Lose sense of control across a transition	Loss of control	Navigating emotional turmoil and self-development	Emotional navigation and coping
…I no doubt felt like it became a little like, like … identity crisis…that like … I didn’t perhaps really know…who I was…that it was like everything was ripped up at the same time … friends…and family … study location … the town…and everything…and it was so mentally pretty difficult to try like in the middle of it all [to] find … find everything new then…it was like that you sometimes didn’t even find your way home when you went on a walk…	It became an identity crisis where I didn’t know who I was, and my friends, family, study location, and town changed at the same time.	Identity crisis due to various changes	Navigating emotional turmoil and self-development	Emotional navigation and coping

Qualitative criteria for rigor were met, including credibility, transferability, dependability, and confirmability (Ahmed, 2024; Lincoln & Guba, 1986). For an overview of the strategies used, see Table III.

**Table III.** Overview of the strategies used to ensure trustworthiness.

Rigor component	Purpose	Original strategies	Strategies used in the study
Credibility	To ensure that the results are accurate, trustworthy, and convincing as perceived by the participants.	Reflexivity: awareness of biases and objectivity during data collection, analysis, interpretation. Triangulation: employment of multiple data sources to verify findings.	Open ended questions, non-leading questions. Interview guide and analysis conducted with co-authors. Interviews audio recorded, observation of non-verbal cues, note-taking during interviews, multiple theoretical perspectives.
Transferability	To enhance the extent to which the results can be applied or adapted to other contexts or settings.	Thick descriptive data: detailed contextual information to assess transferability of findings. Sampling strategies: articulation of sampling process, criteria.	Use of COREQ checklist to adequately describe the setting, sampling, characteristics of participants, methods, procedures, etc. Purposeful sampling to form warranted sample. Reporting of sampling process and criteria.
Dependability	The consistency and stability of research findings over time.	Methodological documentation: detailed documentation of research procedures.	Detailed documentation of the research process at each step. Use of the COREQ checklist to report.
Confirmability	To ensure that the findings are unaffected by the researchers’ biases.	Peer debriefing: engaging with experts or colleagues to avoid bias.	Discussion of the study process and feedback from all co-authors.

### Ethical considerations

The ethical guidelines of the Finnish National Advisory Board on Research Ethics TENK (2023) were followed. Participants were informed about the study purpose, process, their ethical rights, and that they could withdraw at any time. Information was provided twice, in written format when interest to participate in the study was given and orally prior to interview. Interviews were recorded and transcribed verbatim. Personal data were anonymized during transcription; audio files were deleted afterwards. Ethical approval was obtained from the research institution’s ethics committee, and participants signed a consent form. To manage eventual distress during the interview, the participants were informed that they could decline to answer certain questions and that breaks during the interview were allowed as needed. The participants were also informed that they could contact the researchers after the interview if they needed help coping with eventual distress.

## Results

**Figure 1.**
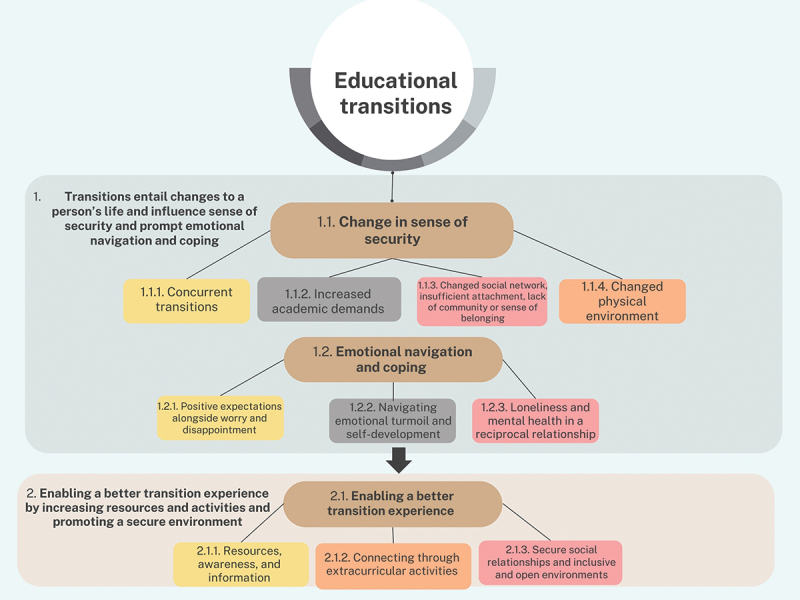
Overview of data analysis.

The data were analysed using qualitative content analysis inspired by Graneheim and Lundman (2004). Two themes, three main categories, and 10 subcategories were revealed. For an overview of the data analysis, see Figure 1.

## Theme 1. Transitions entail changes to a person’s life and influence sense of security and prompt emotional navigation and coping

The first main theme encompassed emerging adults’ experiences of concurrent transitions, including how educational transitions influence their sense of security academically, socially, physically, and environmentally as well as prompt emotional navigation and coping.

### Main category 1.1. Change in sense of security

The participants perceived a change in their sense of security across the transition. Many experienced concurrent transitions, increased academic demands, changed social network, insufficient attachment, a lack of community or sense of belonging, and a changed physical environment, all of which contributed to increased loneliness and decreased mental health.

#### Subcategory 1.1.1. Concurrent transitions

Concurrent transitions could lead to loneliness, anxiety, and stress. Some participants reported that they also faced other challenges during their educational transition, e.g., the end of a romantic relationship, the death of a parent or pet, a friendship ending, a friend’s illness, or changes in a parent’s job.

In the beginning of that year then also my mother died … and it was of course … a pretty traumatic event … and I was then still pretty young…only 19 years [old] … and then like these huge changes kept happening… you became … umm … the last day of in-class lectures came…and you wouldn’t go to the school anymore…then came the stress with all [final exams] and of course you must do well…then you graduated … it was a big goodbye… you’re expected to be so excited for the future and you felt really bad…and you didn’t want to like … think of something … future-related … instead you were like just depressed …. (P5)

#### Subcategory 1.1.2. Increased academic demands

Most participants faced increased academic demands across the transition, including stricter requirements and a faster pace. This increased individuals’ stress, performance anxiety, or general anxiety and affected some participants’ physical health. Some attributed their stress to receiving vague information about academic expectations.

Yeeaaah then absolutely … I don’t know if it affected my feelings of loneliness at all but [it] absolutely no doubt affected my mental health…that I became too stressed then for a period…and got heart palpitations and … difficult to sleep…and all that classical … symptoms of burn-out…and such … so yeah … it was no doubt … really tough …. (P9)

However, some participants perceived that academic responsibilities offered them a positive and meaningful focus.

It didn’t in that way contribute to [my] loneliness because … in one way shall we also say…that if we had some assignments to do then I also had in a way something to do…that it didn’t become that you didn’t have something to do … that there you still had something meaningful to do…to make time go…also perhaps makes one feel good…because you do something you want to do and you manage it pretty well …. (P6)

#### Subcategory 1.1.3. Changed social network, insufficient attachment, lack of community or sense of belonging

The participants generally perceived that changes in their social network, insufficient attachment with new people, or a lack of community or sense of belonging could increase loneliness and decrease their mental health. Some described how moving to a new class in upper secondary/high school led to loneliness due to separation from previous friends or lack of close friends in their new class. Others noted that transitioning to higher education often involved moving to a new location, leading to separation from friends, a romantic partner, or family.

Participants reported that changes in their social network led to feelings of loneliness, anxiety, or emptiness. Some found making new friends and building a community stressful and draining, while others struggled to integrate into new social networks or lost contact with previous friends. Many also experienced physical loneliness from living alone for the first time, which they perceived contributed to loneliness and anxiety.

Well definitely this that some relationships then end … and then always this you don’t have any camaraderie … you don’t have any group … that you’re very lonely … and you don’t have the same relationships … when you move to a new town then … I don’t meet these same people … meet these same friends…that I must like build new relationships…and at this age then I can feel like it can be hard…that I must think … do I fit in here … do I not fit in here … ummm yes…. (P4)

Many participants noted that lacking a community or sense of belonging increased their loneliness. Some found it difficult to form deep connections with new friends, with factors such as differences in age, language, interests, or limited social interactions (small class size) affecting their ability to build such relationships.

But it perhaps is not so much about that you should be like … alone each day … like that you should be physically alone … instead it’s more about that you don’t have anyone that you can feel that you synch with and so on … so that that I could feel at the end of middle school then … that there was some sort of loneliness that I now don’t … and also then a little in the beginning of … studies at [university] then I didn’t really have someone there who … in my course that I feel that I could like … that hey … it feels tremendously good … so you became a little outside [of things]…. (P2)

The participants noted both positive and negative aspects of social media. Social media helped them stay connected with friends, access support, and find a sense of community through groups (e.g., the queer community). Some even met best friends online and connected with people who shared their values and interests.

I have met some of my best friends through social media … that more like these queer communities … that … I’m for example going to a brunch in London to meet like … ehh this whole community this spring … that you … just meet people who have the same values and same interests … then I would actually say that it has impacted positively …. (P7)

However, social media also triggered loneliness and worsened mental health. Some felt that seeing others’ social lives or fun activities, especially on weekends or holidays, led to comparisons. Seeing friends meet up without being invited also increased feelings of loneliness and fear of missing out.

They could have such groups on Snapchat and [have] sent to each other and planned then like for the weekend … or something like that … and that is of course … yeah, I have of course never been part of something like that … I have of course felt really bad and felt very lonely when I have seen … then just like … when everyone gathers … that I have thought then … that now they have met and haven’t invited me …. (P11)

#### Subcategory 1.1.4. Changed physical environment

Many participants revealed that changes in their physical environment could be difficult and negatively influence their loneliness and mental health. For example, some participants observed that adjusting to a new location could be a lengthy process.

The move to [the location for my studies] … for the most part has been such a huge transition because I’ve switched from one daily life to a completely different one…to adapt oneself to a whole new town that I haven’t been to a lot previously … so it has been pretty … it has no doubt impacted me rather a lot…in the beginning I had a lot of anxiety…because it was so unfamiliar everything … and you should get used to it …. (P12)

### Main category 1.2. Emotional navigation and coping

As seen in the second main category, some participants had positive expectations of the transition but could also experience worry and despair. Other participants experienced emotional turmoil but even self-development.

#### Subcategory 1.2.1. Positive expectations alongside worry and disappointment

Some participants expressed excitement and happiness when waiting for the transition; they looked forward to new experiences, a fresh start, and/or becoming more independent. They also hoped the transition would foster new friendships and social opportunities.

Excitement … nervousness … at the same time that I was very confused … and perhaps … anxious to some degree…and for the [first move to the location for my studies] same thing … it was of course … excitement … and … umm … yeah … you were of course … very happy …. (P4)

Still, some participants noted that alongside positive expectations they also experienced worry, nervousness, confusion, or a fear of being alone. While some saw a chance to form new friendships or fit in or had positive expectations about their studies or student life, reality sometimes did not meet expectations, which could lead to disappointment.

When high school started then I thought that … but perhaps it now can be good … that … uhhhh … that yeah … it’s perhaps not that great a change on a deeper level…but new people still…and still … a new beginning … ummm … then it perhaps didn’t become as good as I had thought…that I was … still pretty lonely and alone … even though there were a lot of new people … and … like … still friends at school and stuff but that … that like that you had heard that high school is so fun … that like … a little perhaps like such a … disappointment …. (P10)

#### Subcategory 1.2.2. Navigating emotional turmoil and self-development

Many participants experienced emotional turmoil during the transition. Some experienced shock or a feeling of chaos, while others found the effort to form new friendships emotionally draining. Balancing studies with non-academic activities was challenging for some, and others experienced a sense of not recognizing themselves due to depression. The various changes that occurred encompassed many different domains and led to perceptions of a loss of control, a decline in self-confidence, or even an identity crisis. Some participants isolated themselves due to social burnout, a lack of energy, or physical nausea, which they attributed to the significant changes part of the transition.

The participants noted that changes during a transition, such as moving to a new location, starting a new school, or shifts in social networks, could lead to a sense of a loss of control, decreased self-confidence, an identity crisis, or other major crisis.

The move to [the location for my studies at university] then was no doubt … a second hit … that like…I no doubt felt like it became a little like, like … identity crisis…that like … I didn’t perhaps really know…who I was…that it perhaps became a little … I don’t know … it became a little like … what the fuck moment again…that it was like everything was ripped up at the same time … friends…and family … study location … the town…and everything…ummm … and I don’t know it was no doubt really hard…and it was so mentally pretty difficult to try like in the middle of it all [to] find … find everything new then…it was like that you sometimes didn’t even find your way home when you went on a walk…*laughing*.I think just that identity crisis just … that you were like … that who am I even…and then you didn’t have friends there…who have been a big part of [your] identity…and … family…ummm … yeah just … the question of identity. (P7)

Some participants even perceived that the transition could result in a decline in self-confidence, which they attributed to the various changes they were experiencing.

I had like good self-confidence after that … it had gone well … and I had like trained a lot…so I thought that … in some way then you had like that feeling that…okay … that I will of course like … I had such tremendous self-confidence in some way when I went there…and then it was … in a few weeks … or after some weeks it was totally … gone all of a sudden. (P2)

Some described isolating themselves due to feeling overwhelmed by multiple changes or a lack of energy from meeting new people. Others reported experiencing “social burnout” or physical nausea, which could lead to further difficulties in social relationships.

So it felt so…when I was here in [the location for my studies] … I had no desire to be with people…I just wanted to be in my room and sleep…play some [video] games…and it was almost like I felt physically nauseous …. (P8)

I think it was the summer between lower secondary/middle school and upper secondary/high school that … I isolated myself … like nearly all the time…and I didn’t go to anything … then I had of course become separated from everyone then…and then … when high school started … then I felt that I have no friends … and who should I … search for …. (P11)

Nevertheless, some participants experienced that the transition brought valuable insights and enhanced self-development. They believed that hardships experienced during the transition could eventually lead to personal development, improved emotional management, increased self-security, the development of a clearer identity, or increased confidence.

Now that I have moved to a new town then I feel that I have grown enormously in myself…because it’s been … from going from this “hamster wheel” to a new town…and new things … then you must of course challenge yourself and do things then … it feels like I’ve learned things both socially and about myself … I have learned to know what I myself like and don’t like … and … like that …. (P12)

#### Subcategory 1.2.3. Loneliness and mental health in a reciprocal relationship

All participants noted a link between loneliness and mental health issues. They described feeling lonely not only when physically alone but even in groups or with friends. Some perceived that loneliness stemmed from a lack of attachment, while others linked it to difficulty finding people who could relate to their experiences (specific situations/problems).

The participants highlighted how social factors (relationships, belonging, community, hobbies), balanced daily life (routines, recovery time, own time, demands), satisfaction, meaningful daily life, physical activity, economic stability, and low stress could contribute to good mental health. Some linked mental health issues to general dysphoria, psychiatric diagnoses, or low energy affecting daily life. Many felt that loneliness could lead to mental health issues and/or vice versa, with loneliness and isolation often worsening mental health and leading to further social isolation

They almost…influence each other…if you feel enormously lonely then you can start to develop depression … or anxiety…or if you have some depression from before then you can feel lonely…it’s a little like…they would be in a parasitic relationship … that they feed each other …. (P8)

If you feel bad then you don’t have the energy to be social…and then you make no friends … and then you become depressed because of that as well…and then so … it’s an evil spiral … so it is so easy to say that…yeah well go to lectures…and meet people…go to lunch…and have fun and like that … but it’s not of course just so simple …. (P5)

## Theme 2. Enabling a better transition experience by increasing resources and activities and promoting a secure environment

The second theme encompassed how students can be better supported during educational transitions. The need for accessible resources, increased awareness, available information, and opportunities to connect through extracurricular activities were emphasized. Secure social relationships and inclusive, open environments were also highlighted.

### Main category 2.1. Enabling a better transition experience

The participants felt that the availability of or access to resources, increased awareness, extracurricular activities promoting connection, secure relationships, and inclusive and open environments were important for alleviating loneliness and supporting mental health across educational transitions.

#### Subcategory 2.1.1. Resources, awareness, and information

Most participants reported participating in counselling, therapy, or school curator services, emphasizing their importance and the insights gained. Some suggested that school mental health check-ups could be valuable resources for managing loneliness and mental health.

I don’t know … maybe some type of … checkup … now not any checkup … but some type of discussion with new students … that you feel … yourself … like that … it could just that how you’re doing … and look like … get some advice or something like … referral to that you can do something … just in case you could have some worse type of mental ill-health that it would help to be in touch with just some health care … or generally just check … that is everything okay … how do you adapt … like that … that I think probably like that … perhaps not only people who feel alone … but that students overall … that it would probably do good in that way … that you in that way check that everything is okay in that way …. (P6)

The participants stressed the importance of making mental health service information easily accessible and clear for students, e.g., availability, types of services, where to seek help. They also emphasized the value of transition-related workshops, increased knowledge and information about educational transitions, and counselling. Some suggested that preparation before an educational transition, such as visiting the school or receiving information about school activities, location and surroundings, could be helpful.

This place you are in previously … for example in high school then you would have someone … that what does this mean … how it is to live alone … or something like that … perhaps just this that you … just a little prepare more … just in a more … general way … that it could help just those who perhaps know that they maybe will have difficulties with transitions …. (P6)

#### Subcategory 2.1.2. Connecting through extracurricular activities

Most participants mentioned that activities, events, support groups, and hobbies helped them during the transition. They viewed student associations as a fundamental source of support in meeting classmates, making friends, and finding a sense of community.

You could perhaps find young people who are in the same situation as you … who understand each other…and maybe you develop some kind of community from it … and you could become friends…and … you would also probably feel better because you form new friendships from it …. (P8)

#### Subcategory 2.1.3. Secure social relationships and inclusive and open environments

The participants noted that sharing feelings with partners and friends or support from close relationships helped protect against loneliness. They also emphasized the importance of teachers’/instructors’ support and encouragement in academic matters and in reducing academic stress and anxiety, with some highlighting the need for students to feel comfortable reaching out to teachers for help with loneliness or mental health.

I think it would [have] been more challenging [without my partner] … and then I would for real have been a lot more lonely … but now you have of course still always had someone … and especially if you have been sad then they have always of course been able to … be there and comfort you … but if you would have been alone then … I don’t know where you could turn to then …. (P9)

The participants emphasized the importance of teachers’/instructors’ inclusive leadership, suggesting that randomly assigning groups for group projects could prevent exclusion. Many also highlighted the need for open, safe, and inclusive environments, where peers are open, welcoming and everyone feels included, to help alleviate loneliness and mental health issues across educational transitions.

… in a dreamworld then people would of course … especially young people [would] be more open … to other people … that I think that there are a lot of prejudices … and I think there are … like people are fast to judge … because of different factors … so if people would be more open … to get to know new people …. (P4)

## Discussion

In this study we explored the link between educational transitions and emerging adults’ loneliness and mental health and how they perceive they can alleviate and/or improve these issues. From the results, we discerned that emerging adults’ loneliness and mental health across educational transitions are influenced by various changes in their sense of security, which prompted the participants to go through a period of emotional navigation and coping. The participants also described how a better transition could be enabled.

The participants perceived that concurrent transitions made educational transitions more challenging, leading to increased loneliness, anxiety, and stress. Other researchers, such as Schumacher and Meleis (1994), have found transitions to be complex, with multiple transitions often occurring simultaneously. Another change evident across educational transitions was the increase in academic demands, which heightened stress and performance anxiety, while uncertainty about academic expectations further amplified the stress. However, our results also suggest that academic responsibilities offered some a positive and meaningful focus. In previous studies on upper secondary/high school students, researchers have shown a link between academic stress and mental health, with more productive students reporting better well-being (Subramani & Kadhiravan, 2017). Academic demands can also heighten anxiety, stress, feelings of incompetence or fear of failure (Wrench et al., 2012). Moreover, the transition to higher education often requires greater self-management, which many students may find challenging, leading to stress and pressure to perform (Thompson et al., 2021). Some feel unprepared for the demands of higher education, increasing stress (Thompson et al., 2021; Wrench et al., 2012), while others experience added pressure to achieve high grades (Thompson et al., 2021). This is in line with our results, yet we even discerned that academic demands might also offer a positive and meaningful focus for some students. We recommend providing clear instructions and increased teacher support at the start of the academic year. Moreover, assignments should be better aligned with the type of work students will encounter in the new school or institution.

We found that increased loneliness and decreased mental health across educational transitions were linked to changes in social networks, insufficient attachment to new people, or a lack of community and belonging. The participants reported that leaving old friendships caused loneliness, anxiety, and a feeling of emptiness and that making new friends was stressful and draining. Some struggled to integrate into new social networks and being physically alone for the first time heightened their loneliness and anxiety. Insufficient attachment to new people or a lack of community and belonging in a new setting also increased loneliness, anxiety, and ill-being. The participants noted that factors such as age, language, interests, or limited interactions hindered forming deeper connections. Social media helped them stay connected, find support, and build communities, allowing some to meet new friends online and connect with like-minded people. However, social media also triggered loneliness, fuelling comparisons and increased feelings of exclusion and fear of missing out when seeing friends interact without them.

Other researchers have found that adolescence and emerging adulthood constitute a period when peer groups and intimate relationships become more important (Qualter et al., 2015). At the same time, educational transitions are key periods for forming friendships and social habits (Kirwan et al., 2023). Yet transitions often involve a loss of social networks, social support, familiar objects (Meleis, 2010), and disruptions in relationships and daily life (Kralik et al., 2006). This can affect relationship quality and make forming new connections difficult, making educational transitions a vulnerable period for emerging adults, potentially leading to loneliness (Kirwan et al., 2023). Furthermore, educational transitions usually involve other changes, such as encountering new peers, different learning structures, lower social status (Blakemore & Mills, 2014), moving from familiar to unfamiliar contexts (Benner, 2011), shifting schools, encountering new teachers, and/or larger class sizes (Eccles & Roeser, 2011). Such shifts can lead to disruptions in social networks, introduce new demands (Mikal et al., 2013), and/or lead to concerns about social acceptance (Hanewald, 2013). Successful transitions require integrating into new social networks and maintaining strong relationships (Kralik et al., 2006; Schumacher & Meleis, 1994). Accordingly, interventions should include a focus on preventing disruptions to existing relationships and the fostering of new ones (Schumacher & Meleis, 1994).

Other researchers even suggest that upper secondary/high school transitions can lead to increased loneliness and depressive symptoms due to declining friend support and school belonging (Benner et al., 2017). Students without support from classmates, teachers, or a sense of community also seem to be at higher risk for mental health issues (Santini et al., 2021). Higher education students who move away from friends to their own apartment can struggle to connect with others (Vaarala et al., 2013), lack close relationships (Rönkä et al., 2018), or experience difficulties in communication or a lack of social connections and might experience heightened loneliness (Fardghassemi et al., 2022). Social media can have a positive effect on mental health as it offers opportunities for social connections and peer support, but it might also affect mood negatively, increase loneliness or fear of missing out, and/or decrease life satisfaction and subjective well-being (Zsila & Reyes, 2023). Social media platforms can help higher education students maintain existing friendships and reduce loneliness, yet excessive social media use can heighten feelings of exclusion through exposure to others’ activities online, contributing to a sense of not fitting into group or societal expectations (Käcko et al., 2024). More research should be conducted on how social media affects mental health and loneliness.

We found in this study that a changed physical environment (new town, new school, independent living) could be a difficult adjustment. Students often feel unprepared for independent living and may struggle with challenges such as loneliness (Jaud et al., 2023) or coping when living alone for the first time (Thompson et al., 2021). As seen in previous research, there are differences between emotional loneliness (lack of close attachments) and social loneliness (lack of a network) (Weiss, 1973). Both emotional and social loneliness are common among higher education students, with emotional loneliness being more prevalent (Diehl et al., 2018). Even when surrounded by people, students transitioning to higher education may experience social loneliness due to not knowing enough people or lacking time to form deep friendships (Jaud et al., 2023). From our findings we discerned that the lack of a social network increased social loneliness while the inability to form meaningful relationships in a new environment (the absence of close, personal bonds) increased emotional loneliness. To reduce stress across educational transitions, students should be informed about the adjustments required for independent living.

We found that emerging adults often have positive expectations about educational transitions, including excitement, happiness, and anticipation of new experiences, independence, and friendships. However, they also reported feelings of worry, confusion, fear of being alone, and nervousness. Those with high hopes for friendships, fitting in, or student life sometimes experienced disappointment when reality did not meet their expectations.

Other researchers have seen that students can experience the start of higher education as being an exciting time with many new things to experience (Jaud et al., 2023; Sundqvist et al., 2024). Yet adapting to the demands of higher education can be challenging and some can feel inadequately prepared, which can cause a sense of being overwhelmed (Jaud et al., 2023). Unrealistic expectations of transitions appear to influence the transition experience, and less stress can be associated with a transition if a person knows what to expect (Schumacher & Meleis, 1994). Preparing students with realistic expectations about what a transition can entail might be important in alleviating stress.

We also saw that transitions could lead to emotional turmoil, such as shock, chaos, and the emotional drain of forming new friendships. Some participants struggled to balance studies and other activities or experienced depression. Some experienced a sense of not recognizing themselves due to depression, loss of control, declining self-confidence, or identity crises. Others experienced social burnout, low energy, physical nausea, and/or isolation due to various changes happening at the same time. In Transition Theory it is emphasized that transitions can evoke emotions such as stress, anxiety, depression, or loneliness and are often accompanied by role conflict, low self-esteem, feeling overwhelmed and isolated, or risk aversion (Schumacher & Meleis, 1994). Such shifts can trigger identity reformulation (Meleis et al., 2000) or a loss of self, due to the uncertainty and turmoil following an event or disruption (Kralik et al., 2006).

Yet in this study some participants reported that transition hardships led to personal growth, emotional management, increased self-confidence and self-security, and clearer identity. Transitions are considered to be essentially positive in Transition Theory (Chick & Meleis, 1986). While the mastering of new skills and behaviours is needed to successfully manage new situations or environments (Meleis et al., 2000), growth, self-esteem, empowerment, role mastery, and competence can accompany successful transitions (Schumacher & Meleis, 1994). The finalization of a transition can lead to greater stability than pre-transition (Chick & Meleis, 1986). However, Transitions Theory in relation to educational transitions has been investigated in few studies (Lindmark et al., 2019). This link and how students can be supported so that they can manage and cope with transitional changes more effectively should be examined in future studies.

From the results we saw that there was a reciprocal relationship between loneliness and mental health. The participants described feeling lonely not only when physically alone but also in groups or among friends, often due to a lack of attachment or shared experiences. They identified social factors, balance, satisfaction and meaningfulness in daily life, physical activity, economic stability, and reduced stress as supportive of mental health. Many also noted that loneliness could lead to mental health issues, and vice versa. Some participants attributed mental health issues to general dysphoria, psychiatric diagnoses, or low energy affecting their daily lives.

Our results are in line with other studies where loneliness has been identified as a negative feeling that can be associated with being physically lonely or the feeling of being misunderstood by others (Jaud et al., 2023). Loneliness is a strong predictor of poor mental health among higher education students (Mclntyre et al., 2018). Lonely individuals also more often report moderate or higher psychological distress, depression, impaired self-perceived health (Richard et al., 2017), depressive symptoms (Hutten et al., 2021), suicidal ideation (Beutel et al., 2017), anxiety (Beutel et al., 2017; Hutten et al., 2021; Richardson et al., 2017), stress (Richardson et al., 2017), or somatic symptoms (Hutten et al., 2021). Loneliness and mental health issues might be intertwined. For example, those with mental health issues can isolate themselves from social encounters, which can result in loneliness, and the experience of long-term loneliness can result in mental health issues (Rönkä et al., 2018). As the participants in this study also perceived a reciprocal link between loneliness and mental health, further research into this connection is recommended.

Seen in the results is also that access to resources (counselling, therapy, curator services, school mental health check-ups); increased awareness of available services and where to seek help; and information about transitions (workshops, knowledge, counselling) could help alleviate loneliness and support mental health across educational transitions. Participants noted that preparation, such as visiting or receiving information about a school before transitioning, could be beneficial. In Transition Theory, prior planning and preparation can create more optimal conditions for a transition (Schumacher & Meleis, 1994). Other researchers also emphasize the importance of an introductory week to help students form connections and feel less alone (Jaud et al., 2023). Learning practical skills for independent living, receiving academic support, and getting sufficient transition-related information can help reduce anxiety among higher education students (Cage et al., 2021). Additionally, raising awareness and improving access to counselling and advising services is crucial for students’ mental well-being (Baik et al., 2019).

We found that extracurricular activities, such as events, support groups, hobbies, and student associations, can help reduce loneliness and support mental health across educational transitions. Other researchers have found that involvement in sports and academic activities is linked to more friendships during the upper secondary/high school transition (Bohnert et al., 2013). Researchers noted in a systematic review that fostering social connectedness in group settings could benefit higher education students and alleviate loneliness (Ellard et al., 2023). The role of student councils in organizing events and spaces to help students form connections outside academics is also emphasized (Jaud et al., 2023). Given that students face disruptions to their previous networks and hardships in fostering new ones, increasing opportunities for friendship formation is important.

We discerned the importance of secure social relationships, teacher/instructors’ support, and inclusive environments. Teacher approachability, empathy, and communication are vital for student well-being (Baik et al., 2019), and emotional support from teachers can increase the likelihood of school completion in upper secondary/high school (Tvedt & Bru, 2023). In higher education, mentoring and “buddy” schemes also appear to enhance peer learning, while relationships with tutors and counsellors provide stability (Cage et al., 2021). The role of mentors in easing professional transitions is even highlighted in Transition Theory (Schumacher & Meleis, 1994). Students’ loneliness and academic adjustment across the transition to higher education can even be linked to the social support they receive from family, friends, or others (Sadoughi & Hesampour, 2016). Multiple close relationships and strong ties with parents and friends can reduce loneliness and improve well-being (Calderon Leon et al., 2024). Lack of such support can lead to increased loneliness, isolation, and alienation (Wrench et al., 2012). Given the pressure students often feel in higher education, fostering an inclusive and supportive culture is key to promoting mental health (Baik et al., 2019; Cage et al., 2021). Interventions including a focus on increasing awareness, providing supportive staff, and fostering inclusivity, as well as helping students form new friendships are recommended.

As demonstrated in this study, educational transitions can bring significant changes in sense of security. While educational transitions can foster positive expectations, excitement, and hope for new opportunities, they might also result in a period of emotional navigation and coping, including feelings of shock, chaos, loss of control, identity crises, and decreased self-confidence. Still, for some, this period can serve as an opportunity for personal growth.

We discerned that simultaneous significant changes to sense of security can exacerbate emerging adults’ loneliness and mental health issues across educational transitions. Our findings are in line with Transition Theory, but this theory in relation to educational transitions has been investigated in few studies (Lindmark et al., 2019). While individual factors such as loneliness, stress, depression, or anxiety have been separately explored in previous research, this study contributes to broader and improved understanding of how the simultaneous occurrence of several changes to sense of security across educational transitions exacerbates loneliness and mental health issues. We recommend a further exploration of the subject in light of Transitions Theory and with respect to a proactive approach through which mental health can be supported in future studies. Furthermore, longitudinal qualitative approaches (Kralik et al., 2006) should be employed in future research.

### Strengths and limitations

The results should be considered with respect to the study’s strengths and limitations. A strength of the study is that it explored the experiences of increased loneliness and declining mental health across two educational transitions among Swedish-speaking emerging adults in Finland, a topic that has not been extensively researched before. A qualitative approach was used to obtain more in-depth information, a method that has been underutilized in previous research on the study subject.

Limitations include that the sample primarily consisted of female participants, was ethnically homogenous, and participants with psychiatric diagnoses were included, which may have influenced the results. Including participants with mental health diagnoses could potentially influence accounts of events. However, as the focus of the study was on mental health, valuable personal insights that might otherwise be overlooked have been included. Focus was also placed on emerging adults’ transitions to upper secondary/high school and higher education, but most participants were enrolled in higher education. Although the aim of qualitative research is to uncover in-depth meaning and experience and not generalized findings, the experiences of some groups might not be captured in this study. To ensure more reliable and diverse data and minimize potential bias, participants should ideally have been more diverse. Some interviews were conducted by video conference, which could have influenced the researchers’ ability to capture non-verbal cues. However, the use of video conference also enabled a broader geographical spread of participants, which was desired.

## Conclusion

Transitions entail changes to a person’s life and can influence their sense of security and prompt emotional navigation and coping. Transitions can be associated with positive expectations and self-development but also worry, emotional turmoil, loneliness, and mental health issues. A better transition experience can be enabled by increasing resources and activities and promoting a secure environment. It is important to support the establishment of new friendships, prevent disruptions in relationships, foster a more inclusive environment, and facilitate support and encouragement from teachers/instructors across educational transitions. Preparing students with realistic expectations, information, and knowledge about the transition could also reduce stress. The relationship between these concepts should be further investigated in qualitative longitudinal research.
